# The changing brain: Neuroscience and the enduring import of everyday experience

**DOI:** 10.1177/0963662514521550

**Published:** 2015-10

**Authors:** Martyn Pickersgill, Paul Martin, Sarah Cunningham-Burley

**Affiliations:** University of Edinburgh, UK; University of Sheffield, UK; University of Edinburgh, UK

**Keywords:** bodies and science, discourses of science, lay expertise, patients, popularisation of science, public understanding of science, representations of science, science and popular culture, sociology of health and illness

## Abstract

Discourses of ‘neuroplasticity’ have become increasingly apparent in the neurosciences and wider society. These connect with broader narratives about the ‘changing brain’ throughout the life-course. Here, we explore their presence in the talk of a range of publics. Their presence is indicative of how novel neuroscience is accepted, or not, by our participants. In particular, we suggest that any acceptance of the science relates to their personal and/or professional experiences of change (to their own or others’ subjectivities) rather than to some intrinsic and widely-held significance of scientific concepts per se. Accordingly, we also submit that it is in part through the congruence of some neuroscientific claims to everyday experiences and perspectives that the former are rendered legible and salient. In this respect, ‘lay’ knowledge has considerable import for the wider cultural authorisation of that of ‘experts’.

## 1. Introduction

Over the last decade, the concept of neuro- or brain ‘plasticity’ has become increasingly resonant in international neuroscience research. Whilst scientific discourse is divergent in its deployment of ‘plasticity’, this term can be broadly understood as the potential for “changes in the input of any neural system, or in the targets or demands of its efferent connections, [to] lead to system reorganization that might be demonstrable at the level of behavior, anatomy, and physiology and down to the cellular and molecular levels” (Pascual-Leone et al., 2005: 377–378). The plastic brain is thus framed as a dynamic network, the very nature of which is moulded through subjective experience.

The idea that the developing brain in *childhood* is malleable or plastic has a long history within the neurosciences. Today, studies have been regarded as demonstrating neurogenesis – the growth of new neurones – in animals and adult humans (see Gage, 2002 and references therein). Regarded by some as “a conceptual event (indeed, a conceptual scandal)” (Rees, 2010: 153), the degree to which all areas of the brain can be thought of as plastic is still contentious. Yet, there is a growing scientific consensus that plasticity is a widespread and important feature of the human brain. Within narratives of plasticity, the brain and its environment are taken to mutually shape each other both in childhood and across adult life. This has led to speculation from a range of communities about the degree to which ‘traditional’ distinctions between nature and nurture are dissolving, and new possibilities for both therapy and enhancement opening up (Rubin, 2009).

Within (at least) Europe and North America, a plethora of books, media articles, blogs and other cultural products today draw explicitly or indirectly on the new discourses of brain plasticity in their exhortations to ‘use it or lose it’ and ‘keep your brain fit’. One much discussed technique relating to the idea that the structure and/or function of the brain is changeable is ‘brain training’; this is generally understood as mental exercises that are regarded by some as being able to improve brain functioning. A range of devices, exercises and programmes continue to be promoted as a means of improving memory, enhancing cognition, tackling psychiatric disorders, and combating neurodegeneration (Brenninkmeijer, 2010). Yet, ‘brain training’ has been the subject of much controversy, with many neuroscientists condemning it as unproven and based on an exaggerated understanding of the degree of plasticity the human brain possesses (e.g. Owen et al., 2010). Nevertheless, games designers and science writers (such as Norman Doidge, author of the 2007 book, *The Brain that Changes Itself: Stories of Personal Triumph from the Frontiers of Brain Science*), represent the concept of brain plasticity to wider publics as novel and exciting. In so doing, commentators and the producers of cultural products set out the implications of recent scientific work on brain plasticity for a range of health and social issues, including education, learning, ageing, mental health, sexuality and rehabilitation, commonly situating claims within the language of personal liberation.

Some social scientists and other commentators have already cast their gaze upon these discourses of the changing brain. As Choudhury and McKinney (2013) note, ideas about plasticity have also become intertwined with concerns within civil society about the developing brain and the (over-)use of digital media by children and adolescents. In the UK, where ideas about the changing brain and childhood have collided in social policy agendas (see Allen, 2011), considerable ire has been evoked in some quarters, with accusations being levelled at policymakers that they have been ‘blinded by neuroscience’ (Wastell and White, 2012). In the work of Pitts-Taylor (2010) and Thornton (2011), the concept and popularisation of plasticity in particular has been diagnosed as symptomatic of broader neoliberal agendas that seek to create and order ever more diligent subjects, who can today self-govern at the level of the neurological itself. Other social theoretical accounts of plasticity have underscored the potential of this idea for more liberatory forms of praxis (Malabou, 2008; Papadopoulos, 2011).

It is, however, unclear to what extent ideas about the changing brain are finding traction and salience within the everyday worlds of those who are perhaps likely to be most affected by them. As Wall (2010) shows, related discourses of brain development can evoke responses ranging from anxiety to scepticism in the mothers of young children. Conversely, the work of Elizabeth Fein (2012) has examined situations within which ideas of neurological fixity – not plasticity – structure practice and which can be put to use for various moral and economic ends. This is suggestive of the diversity of public engagements with scientific discourses of brain changes that might be expected (and which has been shown through media analysis with regards to neuroscientific images, for instance; Whiteley, 2012). Drawing on UK focus group data as part of a project on the social and ethical dimensions of neuroscience, we begin here to cast further light on what notions of a changing brain manifest within publics’ discourses, and in what ways. In so doing, we seek to avoid the more “polemical” claims that have been argued to be present within “discussions about the cultural significance of neuroscience” (O’Connor and Joffe, 2013: 256).

Mindful of the cautions of O’Connor and Joffe (2013), and without wishing to take for granted the significance of scientific discourses on the changing brain (even as we recognise its cultural resonance), we thus take analytic cues from a different range of literatures. Firstly, we draw on key ideas from studies of the public understanding of science; in particular, work that has shown the centrality of social identities and social location in directing people’s interest in, reception of, and engagement with scientific knowledge (Kerr et al., 1998; Michael, 1992; Wynne, 1992). Secondly, we find instructive sociological and philosophical investigations around individuals’ embodied experiences of health and illness (Bury, 1991; Busby et al., 1997; Leder, 1990; Monaghan, 1999; Prior, 2003). This research points both to patients’ (shifting) conceptualisations of bodies and biomedicine as illness progresses, and to the distinct understandings of disease and risk that may be evident between physicians and ‘lay’ people. Finally, we are motivated by scholarship around the historical and cultural foundations for the ‘neuroscientific turn’ (Littlefield and Johnson, 2012) that has been documented in various social and epistemic practices (Pickersgill, 2013; Stadler, 2012; Vidal, 2009). Such work is indicative of the degree to which the widely-held ‘novelty’ of neuroscience (including by some social scientists) is in fact itself socially-situated and (co-)produced.

## 2. Methods

This paper draws on data collected for an Economic and Social Research Council funded study aimed at better understanding the place and role of neuroscience in society (Principal Investigator: Cunningham-Burley; Co-Investigators: Pickersgill and Martin). More specifically, the research sought to determine what neuroscientists, patients and professionals who might in some way use neurologic research judged to be the benefits and risks of neuroscience; analyse how moral and ethical valences are ascribed to new social developments occasioned by neuroscientific research; and explore what rights and responsibilities publics perceive themselves and others to have in relationship to the implications of neuroscience. Our aim was to produce reflective research that could contribute to ongoing conversations playing out within sociology and anthropology around science–society relationships (such as those summarised above). As interpretative sociologists, we make suggestions based on and draw reflections from our data, with the goal of producing indicative (rather than definitive) claims that augment sociological theorisation.

Our methodological approach reflected these scholarly aims. We conducted 16 small focus groups with a range of participants (cf. Kerr et al., 1998). This method allows for participants to ask one another questions, further enriching the discussion through the fuller development and substantiation of arguments and positions (and the expression of challenges and ambivalence). Each group contained three to five individuals (with an overall total of 57 participants); the small size of the groups encouraged all members to engage, enabling rich and in-depth discussion. Groups were organised along different biosocial lines (ensuring that a particular social identity was shared by each member of a particular group): patient groups (two from an epilepsy organisation, two from a head injury organisation and one from a dementia organisation), neuroscientists (six), and specific professional groups whose beliefs and practices may be affected by new developments in brain research (based on our knowledge of the professional discourses that we had monitored as part of the wider research project). These were one each of teachers, counsellors, foster care workers, CBT (cognitive behavioural therapy) therapists, and clergy.

As has been shown in other work on biomedicine and society (Haddow et al., 2008; Kerr et al., 1998, 2007), many members of ‘the public’ have diverse interests in and knowledge of science. Recognising this, and mindful of our research questions, we deliberately sought to conduct focus groups in England and Scotland with social groups that might already have an interest in neuroscience. To this end, we also included neuroscientists, with the understanding (based in part on Cunningham-Burley’s experience of researching biomedicine and society) that scientists themselves are ‘publics’ for research that is situated outside their very specific areas of expertise.

Focus groups were recruited via targeted emails to individuals (including patient group staff), and organised either by a key informant or by Pickersgill. Recruitment letters emphasised that the research was a focus group study looking at the social and ethical aspects of neuroscience, and to non-scientists it was stated that prior knowledge of neuroscience was not a pre-requisite for participation. Pickersgill convened all groups, which took place at the University of Edinburgh, at a meeting place such as charitable offices or a community centre (for all patient groups), or at a place of work. Ethics clearance was granted by the University of Edinburgh.

Our focus groups were semi-structured, in order to maximise interaction between members. Questions revolved around rights and responsibilities of neuroscientists, and the import of neuroscience for wider society. Scientists were also queried specifically about how they framed the ethics of neuroscientific research; patients regarding whether they would ever participate in neuroscientific studies; and professionals about any significance neuroscience might hold to their work. The purpose of these questions was to produce dialogue relevant to the research questions that was salient to the particular populations participating in each group. An A4 sheet of paper labelled ‘What is Neuroscience?’ was presented to each participant at the start of the dialogue as a vignette to help foster discussion.

In analysing this discussion data, our approach was twofold. First, we were concerned to examine the data for themes that spoke directly to our research questions (as described above; see Pickersgill et al., 2011; Pickersgill, 2012). Second, a more inductive approach was used to locate themes within the data that were not central to our initial concerns, but which nevertheless appeared salient within the corpus. Transcripts were read multiple times by Pickersgill, and inspected by Martin and Cunningham-Burley. Emergent themes were discussed between the authors, in order to locate ones that should be subject to further analysis. Both Pickersgill and Martin independently identified ‘the changing brain’ (i.e., the focus of this paper) as one such theme. The topic guide did not explicitly focus on ideas about changes in the brain across the life-course or neuroplasticity; talk spontaneously turned to these issues.

Pickersgill attended to this theme through further close readings of the transcripts, followed by manual coding of the material and its organisation into coherent sub-themes using Microsoft Word (which are indicated in the rest of this paper through the sub-headings, and which form the basis for the manuscript). During this process, the transcripts as a whole were continually referred back to, as a means of ensuring that the data and analysis presented here remained grounded in the particular context of the focus groups. This open analytic style thus involves reciprocal relating on the part of the authors between data themes, the data corpus, and wider empirical and conceptual sociological and anthropological literatures. As such, it produces scholarship that interprets the specificities of the data collected but which cannot, of course, be regarded as producing findings that can be taken to be necessarily representative of other publics. The point, as noted above, is to produce sociological statements and conceptual claims, which can then be explored empirically by other investigators for different kinds of populations and issues.

In what follows, and in common with other sociological work in similar areas, we interweave our data and our interpretations of it in order to produce an analytic narrative of focus group discussions of ‘the changing brain’. We conclude with a summation of and reflections on the empirical data and conceptual themes explored and interrogated herein. As will come more sharply into focus as this analysis unfolds, discourses of brain changes, of cerebral development and decline, resonate in important ways with the experiential (i.e., bodily and professional) knowledge of a variety of actors, with implications for our understandings of the societal instantiation of biomedical knowledge regarded as novel.

## 3. The changing brain across the life-course

### Experiences of change

Our participants appeared to view the brain as a “*very, very* complicated thing” (F1, epilepsy group 1) which had complex and multifaceted relationships with behaviour and the rest of the body, and the functions of which could shift. This framing was most strikingly evident in the discussions between individuals suffering from the effects of conditions or events like epilepsy and stroke. These participants commonly discussed their neurological concerns from a life-course perspective, describing, for instance, the onset of dementia or seizures from a particular age, and the ways that brain function and incidences of neurological events changed over a lifetime. As one woman put it, “part of my brain had got worse” (F3, epilepsy group 2) once adulthood was reached.

Negative changes in the brain were related to and evidenced by deficiencies in subjective qualities such as behaviours, interests or skills. For instance, one woman with epilepsy and who had suffered from cerebral aneurisms described how her “organisational skills are nowhere near what they used to be but, then again, they’re much better now than, you know, six, seven years ago” (F1, epilepsy group 1). Thus, whilst the brain might change for the worse, it may also, in time, improve to some extent. This narrative of loss and gain is most evident in the following extract:

I always said to my husband, “You know what,” I says, “I think they’ve moved a creative part of my brain somewhere” and he used to say, “What do you mean?” I used to be artistic, as in drawing, you know, sketching an’, you know, charcoals and things like that, I barely find that I can actually put pencil to paper, in that aspect anymore, but I can write poetry, stories, you know […] [A television programme said that] there can be a shift in the brain. (F1, epilepsy group 2)

Yet, for some respondents, changes to the brain were absolute and wholly negative. One older man, recalling the difference between his life before and after dementia became a problem for him, described how “lots of parts in your behaviour patterns are… changed, some mildly, some quite dramatically” (M1, dementia group). Another participant in this discussion described changes that were gradual, but which had profound effects on subjectivity:

I’m interested in the *change*, because I never *noticed* the change really, […] when it started, the only thing I noticed was I couldnae thread a needle, I couldnae paint or anything like that. (F1, dementia group)

The irreversibility of these changes was difficult to deal with, and necessitated the adoption of psychosocial “strategies” in order to live the best possible life:

I would like to go back to what my life was before, I would love *dearly* to go back to what my life was before, but you can’t do that so you’ve got to say, well either I’m going to make this work for me in some way, I’m going to find some *strategies* in it, or I’m going to accept it and hide behind the net curtains in a kitchen and just stare out the window all day. (M1, dementia group)

On occasion, the origins of brain development and decline were reflected upon. Though remaining embodied, this discourse departed in some ways from contemporary biomedical narratives of plasticity and those embedded within the promotion of ‘brain training’ games; instead, entities such as hormones were figured as drivers of change. As one woman with epilepsy reflected:

We moved […] seven years ago and I started having seizures. I think it’s to do with the time of my life as well because it all started in adolescence, and I’m having hormonal changes again now. (F1, epilepsy group 1)

Another woman described how “during my pregnancy I was seizure free”, but “they came back after I had my son. So there was some. But then every time I took my periods I *always* had seizures” (F3, epilepsy group 1). The third female participant in the discussion likewise pondered “why the brain says ‘no pain, no suffering during pregnancy’” (F2, epilepsy group 1). In the second epilepsy focus group, one of the female participants also linked epilepsy to changes in the brain that were possibly “partially hormonal” (F1, epilepsy group 2) in origin.

In sum, many of our participants in patient groups employed a somatic vocabulary to frame and account for changes in their subjectivities and experience of health and illness over time. We might regard the use of neurological/neuroscientific terms in the talk quoted throughout this sub-section as an example of our participants constructing the science as ‘relevant’ (Schütz, 1946), which in some cases activates an interest in or orientation towards it (which we explore in more detail below).

### The interest of brain science to patients

One of the first things that became clear from the research was that some of our respondents had an interest in neuroscience as a form of entertainment: they watched television programmes, read newspaper articles, and attended public lectures on brain research, and considered that some others might do the same, “Neurology is becoming exciting, it’s become consumable, you know? People want to know about it” (M1, dementia group). When introducing himself to his fellow participants, one focus group member said:

I’m dead interested in, in neuroscience, but more or less how the brain does recover and how people perceive people who have, who have had a head injury, stroke, brain injury. (M2, head injury group)

In this quote, M2 explicitly links his interest in brain science with his understandings of the brain as something that can “recover” – i.e., how it can change. This sub-section focuses on the talk of the minority of patients – and especially this participant – who were familiar with neuroscience prior to the focus groups in order to better explore how (dis)interest in it can be articulated.

Some participants felt that (neurobiological) research that helped to clarify their own experiences of brain changes could be interesting or useful. When asked about the role of neuroscience in treatment for conditions like epilepsy, one participant responded that it should generate a

far deeper and more understanding of how the brain works and functions with regard to itself and the rest of the body, and the chemical changes that take place for various reasons. (F2, epilepsy group 2)

After all, it was “amazing” that “a small change in chemistry” (F3, epilepsy group 2) could have profound effects both on the brain and on the sense of self.

As indicated above, M2 (head injury group) was especially interested in neuroscientific knowledge pertaining to plasticity (and a particularly vocal participant regarding this within the focus group setting). However, for him, acceptance of the claims of scientists did not appear to rest on blind trust in experts so much as emerge from the experiential knowledge of having lived through a traumatic brain injury and noticing the gradual return of function:

Yeah, I mean, I’ve…articles, I went to a lecture, just last week, by this person called Norman Doidge. He’s an American psychologist, and what he’s doing a study on is in brain plasticity, and that’s how the brain reorganises itself. Because I had my, my accident twenty five years ago, and then it was very much one area did something, Broca’s brain, I think it did speech, and if you got that damaged you’d lose your speech; if you lose this bit, you’d lose that bit and I was told that I wouldn’t walk again, or I would for two years, but obviously I am now, and what this lad’s saying is that your lain – your brain! – your brain can be retrained. I mean, to me it’s blatantly obviously, because at one time I was paralysed from the neck down, so obviously, something’s happened up here. I mean, not a lot but, you know, so obviously something, so I’m very much in that thing that, a lot of the things that are coming up now are so obvious that I just don’t know why it hasn’t been us- what’s the word? Looked into more. (M2, head injury group)

Here, lived experience made it “blatantly obvious” that scientific ideas of brain plasticity were valuable in understanding recovery after trauma, and played an important role in validating this knowledge. In other words, science helped make sense of prior experience and provided a framework for understanding the changing body – but science was itself authorised through this experience.

Interest in brain plasticity may, in part, reflect the hopeful aspect of this field of research: what Rubin (2009) might call its ‘therapeutic promise’ (see also Pickersgill, 2011) perhaps encourages a less critical appraisal than findings that imply less positive futures for neurology patients (who do not have a wide range of successful therapeutics on offer to them). Given this, hope of future improvement is very significant and, we suggest, may be part of why Doidge has been so popular. Nonetheless, later in the discussion M2 also distanced himself from the more optimistic tropes of neuroplasticity:

M2: What I think-I think it’s [neuroscience]…it’s very important, you know. But I also think it’s got to be monitored carefully because, I think, like this, this, this person who I went to see, he’s wrote a book called, ‘The Brain that Heals Itself’, and the first couple of chapters are about physical things like, sight, walking, etc., but then he starts going on to mental things, like, your habits, your… everything, I mean, there’s a chapter on pornography and it’s just all about brain plasticity and this person thinks that everything can be *sorted* or has something to do with this brain plasticity, and to me that’s just weird.

Pickersgill: Right, in what sense?

M2: Well, I mean, it’s… it’s just so evangelical! You know, sort of, ‘everything shall be sorted out by this brain that is plastic’, and I’m thinking, but that’s nonsense, you know. As far as I was concerned he was just making up things to, sort of, he said-he was, sort of, like, grabbing something and going, yes it can be done; you know, ‘I hate my dog’; we can cure it with brain plasticity!

M2’s comments became more assertive as the focus group continued, with his final comment (and the final comment of the group discussion) appearing to express a sceptical attitude towards some of the claims he perceived as evident in Doidge’s book: “It’s very interesting at the beginning, it’s incredibly interesting at the beginning, but towards the middle it just goes loonyville!”

We can interpret these statements in at least two, not incommensurable, ways. First, they can be regarded as a form of expectation management, wherein those living with long-term conditions reflexively guard themselves against optimism enjoined by therapeutic promising in order to protect themselves from possible disappointment (on chronic illness and trajectories of expectations, see Bury, 1991). Second, and perhaps more pertinently, they can be understood in terms of the knowledge non-scientists may possess about the institutions of science, including the fallibility of research and the hyping of claims, particularly in popular fora (cf. Kerr et al., 1998; Michael, 1992; Wynne, 1992).

Based on our data – as summarised in this sub-section and that which preceded it – we suggest that changes in the brain throughout the life-course were part of the experiential knowledge some of our participants had about their own bodies (cf. Busby et al., 1997; Monaghan, 1999). Cerebral changes, in both the long and short term, were understood to be generated by some other aspects of the body (i.e., hormones) or even the brain itself: note, for example, F2’s (epilepsy group 1) overall perspective that some part of the brain could self-regulate such that the subjective experience of pain would not be communicated to the embodied self. Participants’ experiential knowledge seemed to rest upon an assumption that the brain is an object of ‘mundane significance’ (Pickersgill et al., 2011) with regards to many, if not all, aspects of health and subjectivity. This has been a key idea within ‘Western’ societies for several centuries, as Vidal (2009) elegantly shows. In some cases, taken-for-granted knowledge was deemed to be evidenced by sources as diverse as television programmes and medication advice; F1 (epilepsy group 1), for instance, described how a doctor informed her that one of the antiepileptic medications would counteract the effect of the contraceptive pill, indicating that “there’s something to do with hormones there. It’s-and especially the way I’ve been since, all my hormones have started going a bit AWOL.” Neurologic research, as encountered through popular media (for instance), may also have had an important explanatory and legitimating function for our participants to make further sense of their personal perspectives. In the next section, we continue this discussion of the changing brain through (primarily) data from our focus groups with different professionals.

## 4. The changing brain in professional practice

### The interest of brain science to professionals

The interest in, and explanatory and legitimatory function of, scientific ideas around the (changing) brain that was evident within the narratives of some patients was also apparent in the focus groups with professionals. Perhaps reflecting recent policy initiatives around children’s brains (Wastell and White, 2012), exposure to and enthusiasm for neuroscience was especially clear within the focus group convened with members of a foster care association. One respondent described attending

[a] training course […] where it spoke about children who suffered severe trauma in early life, that the different parts of their brains don’t wire – my technical terms aren’t as good as F2! – up as a child who hasn’t suffered trauma, and how this impacts on behaviour. (F1, foster care professionals group)

Another discussed lectures and workshops she had been to in this area, framing these as events that had furthered her interest in the brain (and, indeed, stimulated her to accept our invitation to join a focus group). In particular, she recalled a seminar in which the brains of ‘normal’ versus ‘traumatised’ children were shown together, and described how “the power of the brain is just fascinating” (F2, foster care professionals group). Thus, even when the participants were not entirely certain about the information they were presented with, images of “traumatised and neglected” brains had a compelling rhetorical force that appeared to concretise ‘lay’ ideas about cerebral changes, and, following hybridisation with ‘expert’ neuroscientific knowledge, embedded these within professional education. For example, F1 (foster care professionals group) found the courses she attended valuable in that the neuroscientific content

kind of explained it [her professional experiences] a bit better. Because I think sometimes when you’re dealing with these difficult behaviours day in and day out as our carers do, it’s quite easy to forget the reasons behind it, and I thought that was a really nice way of explaining it. (F1, foster care professionals group)

For the individuals taking part in this particular focus group, the opportunities for personal and health improvement that scientific discourses on neuroplasticity implied were especially provocative, suggesting new possibilities that might benefit a variety of individuals:

[T]hat lecture that we went to [gestures to co-worker], the neuroplasticity, it was interesting because it was saying, like, the model that hearing is stored here, and that’s stored there, they’ve tried to kind of map out the brain, and that doesn’t really stand up now because when one part is damaged another part seems to be able to take over the functions. (F2, foster care professionals group)

Counsellors too noted the (potential) utility of neuroscience in enhancing their practice through its empirical claims regarding the negative changes to the brain that might result from traumatic experiences. As one participant described:

[T]he main thing for me really, in the work that I do, is the stuff around trauma and how you know the pathways get broken and […] how you can kind of re-forge those links and re-process trauma and get it put into the right part of the brain. All of that’s useful, I think; and there are useful ways of working with clients that, that are traumatised, and that can really help. (F3, counsellors group)

Research on the changing brain was also judged to be important in that it buttressed the epistemological claims of these professionals against those who might question their expertise. This was valuable for the participants working within foster care, who felt that they were regularly called upon to publicly justify their practice and the expertise structuring it:

[W]hat we deal with in social care which isnae a’ exact science, and people sometimes who don’t work within that sector find it hard to believe, and hard to accept when you’re trying to explain behaviours. (F1, foster care professionals group)

A key mechanism through which neuroscientific knowledge “explained” professional experience was through the situating of older, psychosocial perspectives within a neurobiological framework. Attachment theory, which has origins in psychoanalysis and developmental psychology, is a key example of this:

The way that [the foster care organisation] […] is beginning to organise its post-approval training very much ties up the ideas of trauma and loss, processing and attachment ideas, so our carers are introduced in pre-approval training to, to the idea that brain development will be affected by, by trauma and loss. And certainly I think as a profession we’re much clearer, I think because of some of these images, about the *visible* connection between early, early experiences and behaviour and development. So yeah, and especially we’re much more fluid with the idea now how damaging *neglect* is, for example, whereas ten years ago we thought it was, that we needed much more to be worried about physical and sexual abuse. (M1, foster care professionals group)

Yet, from the focus group discussion, it was not apparent that practice per se had shifted in response to neuroscience, even if the concepts understood to structure and justify it had done. This issue is discernible within an exchange between two participants in the focus group with counsellors:

F1: I suppose what I, what I wonder is that if you don’t know anything about neuroscience is it possible that just the way that you do-the way that I do person-centred counselling, is that alone, you know, if there can be an improvement in the client just through that alone without, without any of this [brain research]?

F3: Absolutely, ’cause when I started working I didn’t know anything about neuroscience. I just did what I do. It was more just kind of learning it as we went along, and holding an awareness […] rather than any kind of specific ‘well, we’re working this way because of that’.

For F3, neuroscience research is “like a framework for thinking about something. But it’s not the whole story.” F1 thought similarly, remarking: “I don’t think neuroscience would ever completely, like take over and be, like, the be all and end all.” The notion that neuroscience is somehow significant, but still marginal, to the concerns of professionals also became clear in the foster care focus group, when one participant noted:

[T]he most useful thing to do is that we spend most of our time supporting carers or working with teachers. Rather than having debates about neuroscience. (M1 foster care professionals group)

The data presented in this sub-section underscores how scientific discourses concerning the changing brain become hybridised and embedded within some kinds of professional learning to help *justify* current theory and practice; it also suggests that neurobiological research is perhaps (and only to some slight extent) *redirecting* the concerns of foster care workers (at least, in the eyes of these participants). As indicated by the excited comments of F2 (foster care professionals group) in response to neuroscientific imagery, the focal power of brain scans as a means of conveying complex neurologic concepts and statistical data through a relatively simple, brightly coloured ‘picture’ might, we suggest, be regarded as having import for any shifts that are occurring (cf. Dumit, 2004). Such images are encountered through meetings, training and the popular media. Nevertheless, in spite of the ‘magnetic appeal’ (Joyce, 2008) of biomedical images and the information they are often deemed to convey, it is not clear that professional interest in the neurological represents a full ‘neuroscientific turn’ (Littlefield and Johnson, 2012). Whilst neurologic perspectives help to frame and justify practice, they are used by those who took part in this research “a little bit more loosely than the kind of formal, formal ideas” (M1, foster care professionals group). Neuroscientific ideas about (for instance) the plasticity of the brain cannot be presumed to translate unproblematically into professional praxis: they must be actively and selectively put to work, and may be reshaped in the process.

### Professional scepticism

As we have seen, ideas about brain changes were important to some of the professionals who participated in this research. Yet, scepticism with regards to knowledge claims was also in evidence. This dialectic between engagement and scepticism was most apparent within discussion about education, and hence especially clear in the focus group conducted with teachers (thus, that dataset is a particular focus of this sub-section).

The teachers who participated in this research commented on how “talk about exercising your brain” (F1, teachers group) was “a bit of a theme at the moment” (this was also noted by various other participants, including some of the scientists). Discourses of changing and improving the brain through computer games and other tools aimed at cognitive enhancement, or preventing cognitive decline, were viewed as “becoming more popular” (F1, teachers group) within not only education but also wider culture. Examples were given of articles in “women’s magazines about keeping your brain active” (F2, teachers group) and special segments on popular television programmes.

For the teachers, the idea that the brain could change through the life-course was a serious business that they were exposed to through educational guidance and recommendations. For instance, they noted guidance received on ‘Brain Gym’ (a controversial programme through which students are encouraged to carry out specific movements in order to enhance cognitive function). Yet, the principles of Brain Gym were not regarded as translating well into classroom practice. As one participant remarked, although some of his colleagues employed Brain Gym techniques, many “people will go away all enthused for a couple of weeks and do things, and then forget about it” (M1, teachers group). This is suggestive of both the compelling nature of ideas connected with the brain, and the degree to which these are not unique but instead merely one of many cultural enchantments that can be experimented with by social actors and then set aside.

Even when teachers feel directed to undertake activities such as Brain Gym in the classroom, doubts about the facility of these to enhance learning through changing and developing neural pathways endured. M1 (teachers group) stated that he had “distanced” himself from it, whilst another participant (F2) in the discussion reflected that she had once read that it had been “decried” as “a load of old twoddle”. Such ‘distance’ can of course be regarded, in part, as a consequence of the everyday exigencies of the workplace, although this is not incommensurable with the possibilities that the neurologic ontology Brain Gym seeks to perform does not resonate with teachers’ own understandings of how individuals learn and change. M1 also constructed ‘distance’ between the professions and academic neuroscientific research. He noted that his most likely contact with neuroscience would be “an article in the Times Educational Supplement for example, which is written about a piece of research which has been reported [in] such and such”. As M1 put it, there “are very few teachers who go out and read all the neuroscience on brains, come back into the classroom and do it. It doesn’t happen that way”.

A participant in one of the focus groups conducted with neuroscientists noted that “all my family are teachers and a lot of people I know are teachers” (F2, neuroscientists group 1) and that “my stuff [research] relates to development in children”. Acting as an informant, she reflected on the topicality of neuroscience within education, but also the challenges to her expertise (which was seen as having relevance to education) and the ‘distance’ between neuroscientific laboratory research and teaching practice that could be constructed:

You know, they’ll have a different view than I do and that’s the case, so I don’t think they see you necessarily as an expert on something that they know nothing about. It’s much more of a two way street whenever I’ve talked to teachers about things. They’re really interested but… Yeah, and I guess a lot of the stuff, at this stage it doesn’t directly apply to them in the classroom so it’s useful to have that, that kind of interaction with them but, yeah, I don’t think they see it as something that’s necessarily kind of directly impacts directly on what they do.

The reaction she reports overlaps closely with the content and nature of the discussion that occurred with the focus group with teachers described above. Such comments do not necessarily reflect professional *resistance* to (neuro)science per se, although they do highlight a lack of time and interest in ideas of brain training, linked to scepticism about its utility (cf. Pickersgill, 2011) and a deliberate positioning of it outside the perimeters of the ‘zones of relevance’ (Schütz, 1946) salient to the participants.

## 5. Conclusion

The emergence of neuroplasticity discourse has been compelling to many who work within biomedicine (Rees, 2010; Rubin, 2009). At the same time, these ideas have found traction within a range of popular media (Pitts-Taylor, 2010; Thornton, 2011). As Prior notes in his work on neurological disorders, “what lay people recognise and report upon is change, and not disease” (2003: 48). The changes that many of the patient participants in this study recognised in themselves (which were often negative, but occasionally positive) sometimes directed their attention towards ‘popular neuroscience’ which appeared to be validated not (solely) through the authoritative voice of scientific experts, but with regard to their embodied, lived experience. New scientific discourses of brain plasticity provided one framework for articulating lived experience, but other biomedical vocabularies (e.g. endocrinological frames) were also employed.

The promissory aspect to scientific findings around plasticity and similar claims regarding the changeable nature of the brain within popular neuroscience (as discussed in the introduction) is reflected in (and perhaps in part produced by) professional activities (e.g. training) experienced by some of our participants, especially those working within education and social care. Here, for instance, neuroplasticity research offers up hope that children who have experienced trauma can be ‘normalised’ through educational and care practices; based on our data and wider research, we suggest that the authorisation for such science comes from care workers’ experience watching such children grow into ‘adjusted’, ‘healthy’ members of society. In the process, older ideas about the psychology of learning and behavioural development appear to be reframed, at least partially, within a neurobiological idiom. This at once legitimises existing practice, whilst also potentially redirecting it (e.g. towards a focus on neglect in foster caring).

Within the data presented we also see traces of scepticism regarding the promise of plasticity. Here and more generally, for patients this may manifest as a careful managing of expectations: hopes formed in order to assert control and certainty over the biographical disruption of serious neurological disorders (Browner and Preloran, 2010) and carefully weighed up against the need for prudence in order to protect against the subjective distress of profound disappointment should expected therapeutic advances fail to materialise. Likewise, for professionals (especially, teachers), experience of everyday work and familiarity with its rhythms, challenges and restrictions may create scepticism and even distrust (cf. Pickersgill, 2011). In this respect, whilst the concept of brain plasticity has been understood as a tool of governance (cf. Thornton, 2011), we regard it here as a window through which to better appreciate how individuals ascribe significance to and construct interest in ‘new’ (scientific) knowledge (particularly as an explanatory framework for extant professional practices and observations, which are then further authenticated in the process).

The data on which our analysis draws is deliberately diverse in order to explore some of the heterogeneity of contemporary engagements with ideas about the brain. Further, as noted in our methodology section, it samples populations who were expected to have some pre-existing interest in neuroscience – and a different sampling strategy might have yielded different results. Yet, based on our data, we can nevertheless make conceptual reflections that can be explored in future work, using a range of case studies. In particular, we suggest that scientific knowledge on its own is insufficient to transform conceptualisations of selfhood or remake professional practice. Perhaps more important is the degree to which research (and its popular instantiations and translations) is congruent with individuals’ everyday experiences. Of course, scientific constructs, such as ideas about the brain, do filter into and shape contemporary culture and potentially provide new ways of explaining experience that might ultimately become taken-for-granted means of comprehending the world and subjective experience. However, for this to occur, we submit that any changes must necessarily emerge from a reciprocal interaction between the lived experience of embodied individuals, existing cultural frames, and prominent scientific discourses. Such processes are complex, and likely do not proceed with the immediacy implied by some of the more hyperbolic claims regarding the intrinsic import of (neuro)science for how we understand our selves.
